# Transcriptional regulation of *SOS1* by CycC1; 1-WRKY75 complex under salt stress

**DOI:** 10.1093/plcell/koad113

**Published:** 2023-04-25

**Authors:** Maryam Rahmati Ishka

**Affiliations:** Assistant Features Editor, The Plant Cell, American Society of Plant Biologists, USA; Boyce Thompson Institute, Ithaca, NY

Soil salinity is a major threat to agricultural productivity. Plants have evolved sophisticated mechanisms to cope with salinity. The Salt Overly Sensitive (SOS) pathway plays an important role in salt tolerance by facilitating the extrusion of excess Na^+^ from cells. The core components of the SOS pathway in Arabidopsis include SOS1, SOS2, and SOS3. A salt-mediated increase in cytosolic calcium is perceived by the SOS3 EF-hand calcium-binding protein, leading to SOS2 activation. SOS2 is a Ser/Thr protein kinase that phosphorylates and activates SOS1, a Na^+^/H^+^ antiporter that facilitates Na^+^ efflux from the cells. How *SOS1* is transcriptionally regulated in response to salt remained elusive. In this issue, **Kai-Kai Lu and colleagues (Lu et al. 2023)** found that C-type Cyclin1; 1 (CycC1; 1) forms a transcriptional repression complex with WRKY75 to downregulate *SOS1*, thus acting as a negative regulator of salt stress response in Arabidopsis (see Figure).

**Figure.**
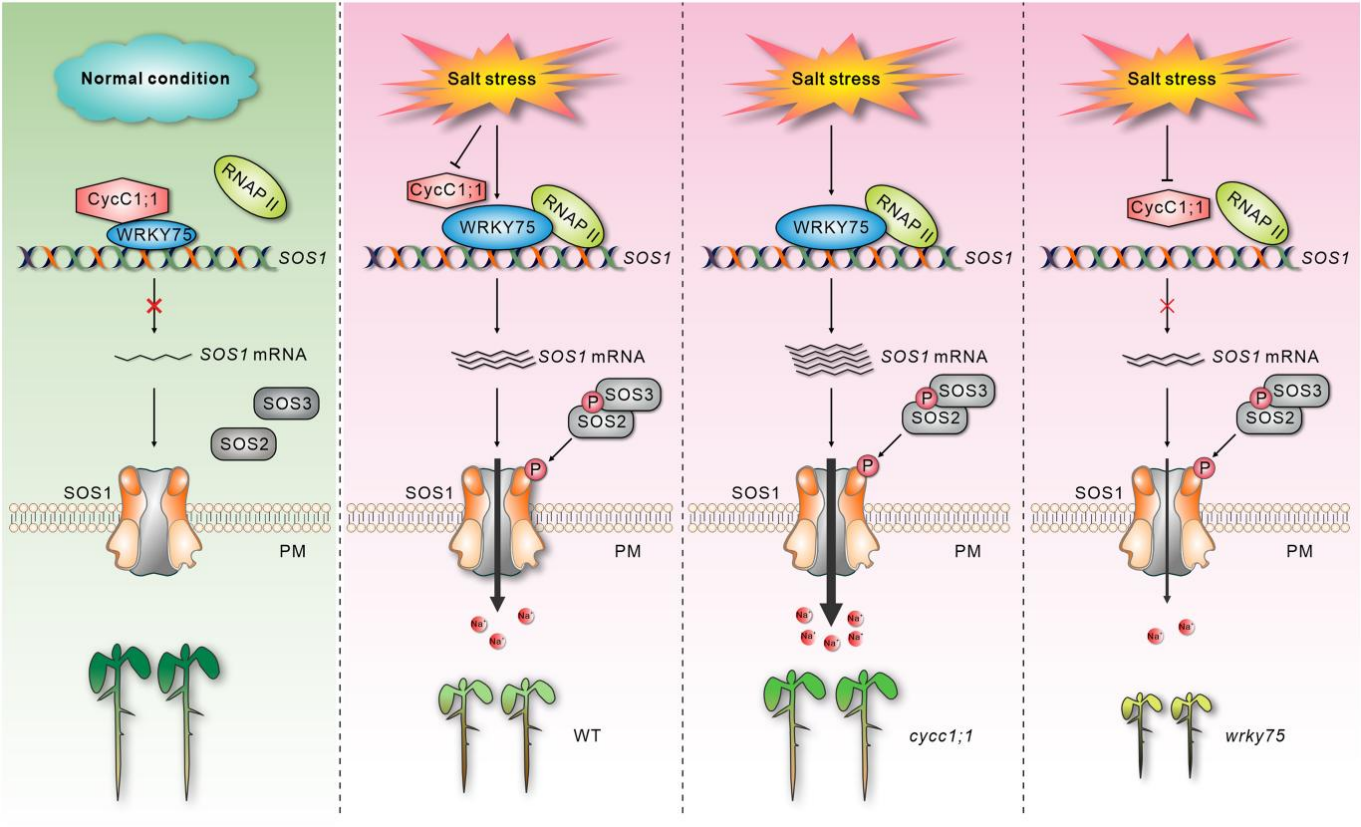
The proposed model describing CycC1; 1-WRKY75 mediated transcriptional regulation of *SOS1*. Under normal conditions, CycC1:1 interacts with WRKY75 to form a transcriptional repression complex that inhibits *SOS1* gene expression. However, salt stress inhibits *CycC1; 1* expression and enhances *WRKY75* expression, leading to increased recruitment of RNAP II to the *SOS1* promoter, activating its expression and enhancing salt tolerance. Adapted from Lu et al. (2023), Figure 8.

CycC1; 1 is a plant mediator protein complex subunit that influences gene transcription by acting as a bridge between transcription factors and RNA polymerase II (RNAP II) (Agrawal et al. 2021). CycC1; 1 is known to play a role in plant immunity by regulating defense-related genes (Zhu et al. 2014). Lu et al. (2023) found that a *cycc1; 1* T-DNA insertion mutant was less sensitive to high salt than wild-type plants as measured by seed germination, cotyledon greening, and seedling growth parameters, suggesting that CycC1; 1 plays a role in the salt stress response. Using gene expression and GUS reporter assays, the authors showed that *CycC1; 1* was expressed mainly in the root stele, similar to *SOS1*, during early developmental stages and that salt treatment decreased its expression, implying that CycC1; 1 acts as a repressor of the salt stress response.

Using a sodium-specific fluorescent dye to measure Na^+^ content in roots, the authors showed that Na^+^ accumulation increased upon salt treatment in *cycc1; 1* mutant and wild-type plants, but the mutant accumulated significantly less Na^+^ than the wild-type plants. Whereas gene expression of all 3 core components of the SOS pathway (*SOS1*, *2*, *3*) was induced upon salt treatment in wild-type plants, *SOS1* expression alone was induced to a much greater extent in the *cycc1; 1* mutant, indicating that CycC1; 1 mainly influences *SOS1* expression and the accumulation of *SOS1* transcripts. Chromatin immunoprecipitation assays showed that CycC1; 1 binds to the *SOS1* promoter. In addition, the amount of RNAP II associated with *SOS1* genomic DNA after salt treatment was greater in *cycc1; 1* than in wild type, suggesting that CycC1; 1 inhibits salt-induced *SOS1* expression by interfering with the RNAP II association to the *SOS1* promoter. The salt sensitivity phenotype of a *cycc1; 1 sos1* double mutant further suggested that CycC1; 1 acts upstream of SOS1 in plant salt stress response.

Next, the authors used yeast 2-hybrid, bimolecular fluorescence complementation, and co-immunoprecipitation assays to identify WRKY75 as a CycC1; 1 interactor and electrophoretic mobility shift assay to show that WRKY75 binds to the W-box motif in the *SOS1* promoter. In contrast to *cycc1; 1* mutant plants, *wrky75* mutants were salt sensitive and showed decreased expression of *SOS1*, indicating that WRKY75 also plays a role in salt-induced *SOS1* expression. The authors also found that *WRKY75* has a similar expression pattern as *CycC1; 1* and *SOS1*, that is, mainly in the root stele. Using a dual LUC reporter assay, the authors found that CycC1; 1 interferes with WRKY75-mediated transcriptional activation of *SOS1*.

Together, Lu et al. (2023) show that CycC1; 1 and WRKY75 form a signaling module that facilitates the dynamic regulation of *SOS1* expression. Under normal conditions, CycC1; 1 blocks WRKY75-mediated transcriptional activation of *SOS1*, whereas *CycC1; 1* expression is suppressed and *WRKY75* expression is upregulated under high salt, promoting *SOS1* expression and leading to enhanced salt tolerance (see Figure). Further work is needed to identify the receptor and signaling components leading to the salt-induced downregulation of *CycC1; 1*. Also of interest, 2 more newly published studies shed light on the SOS2 component of the SOS signaling pathway and its connection to shade avoidance as well as salt tolerance through interaction with phytochrome A and B light receptors (Han et al. 2023; Ma et al. 2023). Thus the SOS pathway is emerging as a signaling hub in plant response to a variety of environmental conditions and stresses.

## References

[koad113-B1] Agrawal R , Jiří F, Thakur JK. The kinase module of the mediator complex: an important signalling processor for the development and survival of plants. J Exp Bot. 2021:72(2):224–240. 10.1093/jxb/eraa439 32945869

[koad113-B2] Han R , Ma L, Lv Y, Qi L, Peng J, Li H, Zhou Y, Song P, Duan J, Li J, et al The salt tolerance regulator SALT OVERLY SENSITIVE2 promotes shade avoidance by stabilizing the growth-promoting factors PIF4 and PIF5. Plant Cell. 2023. 10.1093/plcell/koad119 PMC10396385 37119311

[koad113-B3] Lu KK , Song RF, Guo JX, Zhang Y, Zuo JX, Chen HH, Liao CY, Hu XY, Ren F, Lu YT, et al Cycc1; 1-WRKY75 complex-mediated transcriptional regulation of *SOS1* controls salt stress tolerance in *Arabidopsis*. Plant Cell. 2023:35(7):2570–2591. 10.1093/plcell/koad105 PMC10291036 37040621

[koad113-B4] Ma L , Han R, Yang Y, Liu X, Li H, Zhao X, Li J, Fu H, Huo Y, Sun L, et al Phytochromes promote plant salt tolerance by enhancing SOS2-mediated phosphorylation and degradation of PIF1 and PIF3 in the light. Plant Cell. 2023. 10.1093/plcell/koad117 PMC10396371 37119239

[koad113-B5] Zhu Y , Schluttenhoffer CM, Wang P, Fu F, Thimmapuram J, Zhu J-K, Lee SY, Yun D-J, Mengiste T. CYCLIN-DEPENDENT KINASE8 differentially regulates plant immunity to fungal pathogens through kinase-dependent and-independent functions in Arabidopsis. Plant Cell. 2014:26(10):4149–4170. 10.1105/tpc.114.128611 25281690 PMC4247566

